# State Government Policy Responses to the COVID-19 Pandemic in the United States 2020–2022: Concordant Heterogeneity?

**DOI:** 10.18103/mra.v11i4.3693

**Published:** 2023-04

**Authors:** James A. Koziol, Jan E. Schnitzer

**Affiliations:** 1 Proteogenomics Research Institute for Systems Medicine, La Jolla, California 92037, USA.

**Keywords:** governmental intervention, public health policy, COVID-19 mortality

## Abstract

**Objectives.:**

We investigate governmental responses to the COVID-19 pandemic on a statewide basis between January 2020 and June 2022, together with mortality rates attributable to COVID-19 over the same period. Our aim is to demarcate the states’ responses, and examine whether these differential responses are associated with COVID-19 mortality.

**Methods.:**

Our study is based on individual state data from the Oxford COVID-19 Government Response Tracker, OxCGRT. We focus on the Government Response Index, the most comprehensive index tracked in the OxCGRT dataset. We use multivariate techniques to group the states into clusters relative to their similarities on the Government Response Index, and determine mortality rates attributable to COVID-19 in the individual groups.

**Results.:**

We find that the Government Response Index was sustained at relatively constant levels in the states, with two major transition periods: a rapid rise in stringency during April through June of 2020, and a gradual decline in May and June of 2021. Heterogeneity in the Government Response Index dramatically increased in 2022. No consistent patterns emerge when relating government stringency measures with COVID-19 mortality rates.

**Conclusions.:**

There is inconsistent evidence that increased governmental stringency is associated with lower COVID-19 mortality; judicious selection of time frames can lead to contrasting inferences. Political trends and motivations appear to have an outsized influence on governmental responses to the COVID-19 public health crisis, to the detriment of the populace.

## INTRODUCTION

The Oxford COVID-19 Government Response Tracker, OxCGRT^1^, contains a standardized set of composite indices that measure governments’ responses to the impact of COVID-19 in their respective jurisdictions. Utilizing this database, we here investigate states’ responses to the ongoing COVID-19 pandemic in the United States, over the period January 2020 through June 2022. We examine mortality rates attributable to COVID-19 in the individual states, in association with government intervention and policy actions as reflected in the OxCGRT indices. Our aim is to inform and improve interventions and policy choices to the COVID-19 pandemic, if shown to be effective relative to mortality.

Although our focus is solely on the United States, implications of our findings extend beyond these borders, impacting political accountability and governance related to public health policy.

## METHODS

We downloaded the complete Oxford dataset^2^, and abstracted United States data from the individual states and the District of Columbia (DC) over the period 1 January 2020 through 30 June 2022. For each state and the District of Columbia (DC), the data consist of daily scores on the policy index of our focus, the Government Response Index (GRI). The composition of this index is described in detail by Hale and colleagues^2^; briefly, the Government Response Index is the most comprehensive index tracked in the OxCGRT dataset, comprising closures and containment measures restricting people’s behaviors (e.g., school and workplace closings, restrictions on gatherings, stay-at-home requirements, international travel controls), and also incorporating economic measures (e.g., income support or debt relief) and health measures (e.g., testing policies, contact tracing, facial coverings, vaccination policies, protection of the elderly). The index is numerical, higher values indicative of increased stringency or imposition of government strictures. See also the OxCGRT website^3^ for relevant methodology utilized in calculating the index.

We divided the 2020–2022 data into 5 consecutive subsets, each spanning 6 months. Within each of the subsets, we undertook a hierarchical cluster analysis of the states and the District of Columbia relative to their daily scores on the GRI. We chose the Ward method of clustering^4^, and the Minkowski metric (with parameter p=1; also known as Manhattan distance) for initially determining similarities between the states relative to the GRI. From each resulting dendrogram, we amalgamated the states on the basis of their mutual similarity into four groups or clusters for further assessment.

For each of the five {year, period} subsets, we plotted group means of the daily scores on the GRI for the four groups determined from the cluster algorithm, the purpose being to establish the degree of separation of the four groups relative to their scores on the index. We determined mortality rates attributable to COVID-19 for each state over each {year, period} subset from the mortality data available from the Centers for Disease Control^5^, together with state population data from the US Census^6^. In this endeavor, we calculated mortality rates separately for individuals 18 to 64 years old and individuals 65 years and older. We then undertook Poisson regressions with group membership (as established from the cluster amalgamation) as the putative predictor of mortality rates during each {year, period} subset, and separately for the two age cohorts. From these regressions, we abstracted estimated mortality rates and associated 95% confidence intervals for each group. These are given in tables underneath the choropleths illustrating group membership during each {year, period} subset.

We also used Poisson regressions in a further exploratory analysis, comparing mortality rates between two subsets of states, those that were consistently in the highest (most restrictive) two tiers of groups or clusters established from the states’ daily GRI indices over the 2020 through 2021 {year, period} pairings, and those that were consistently in the lowest (least restrictive) two tiers. For comparative purposes, we replicated these analyses over the January 2020 through December 2021 time frame. Note that in this instance, mortality rates correspond to a two year time period.

## RESULTS

**Figure 1.**
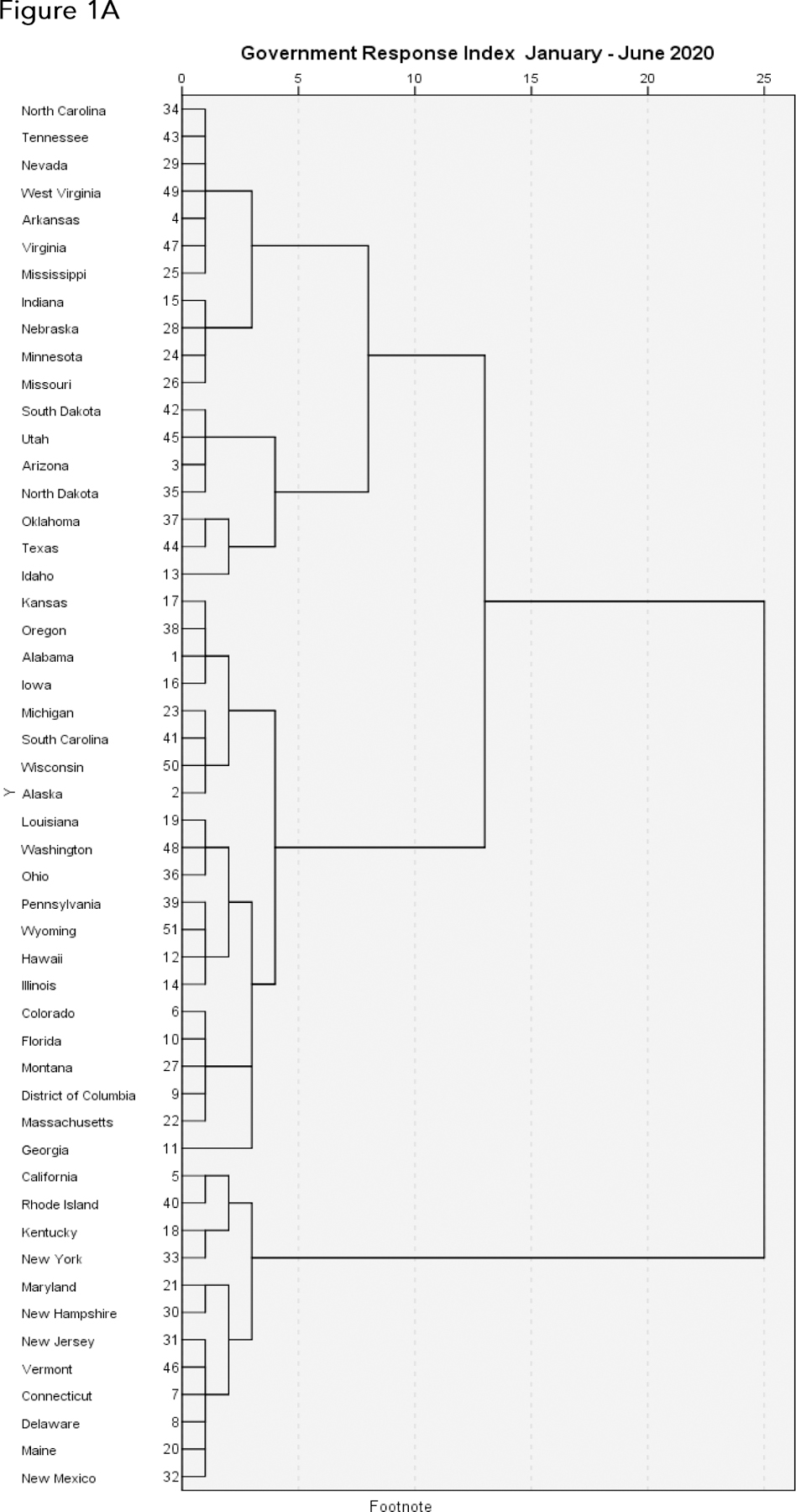

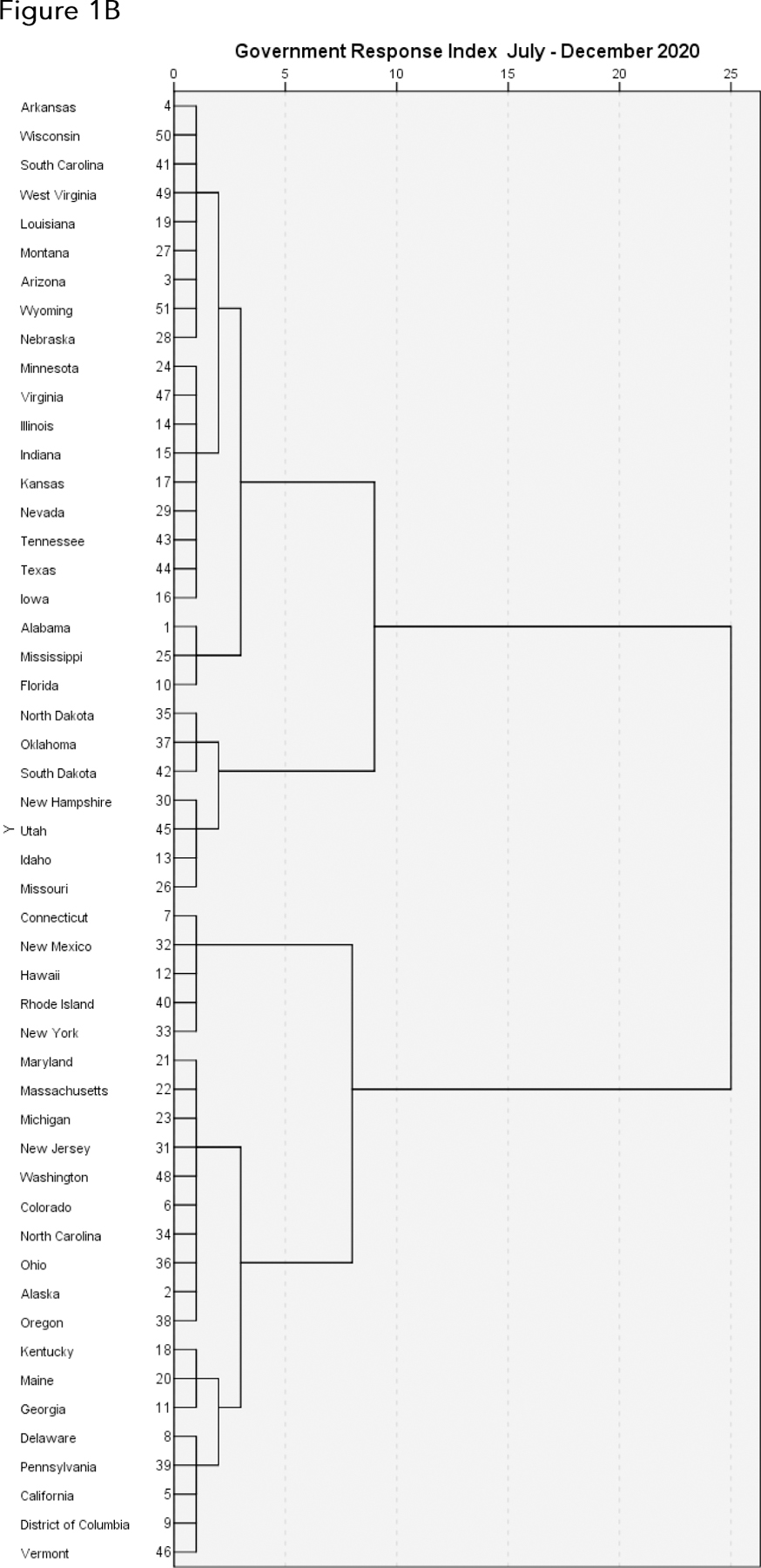

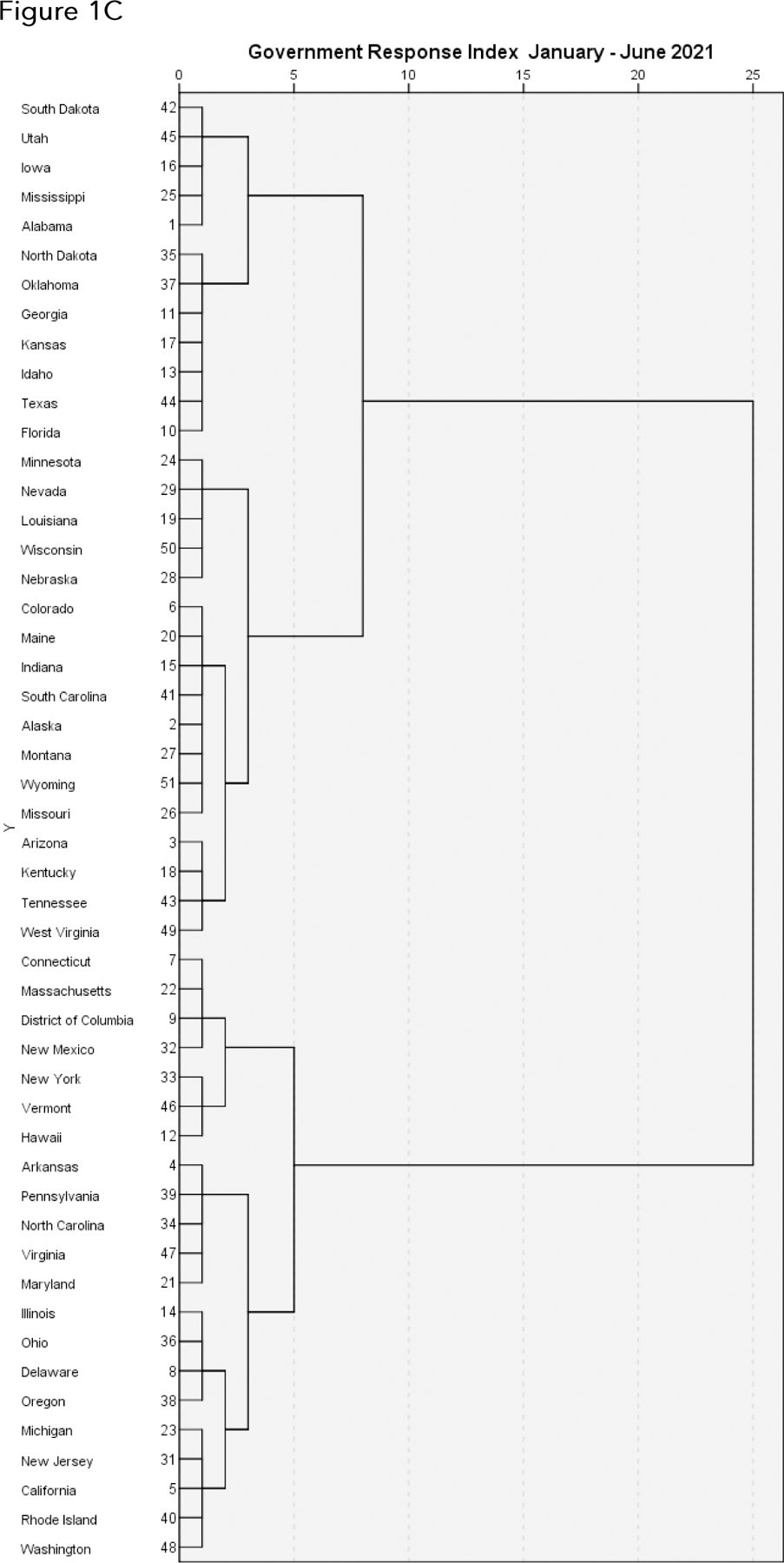

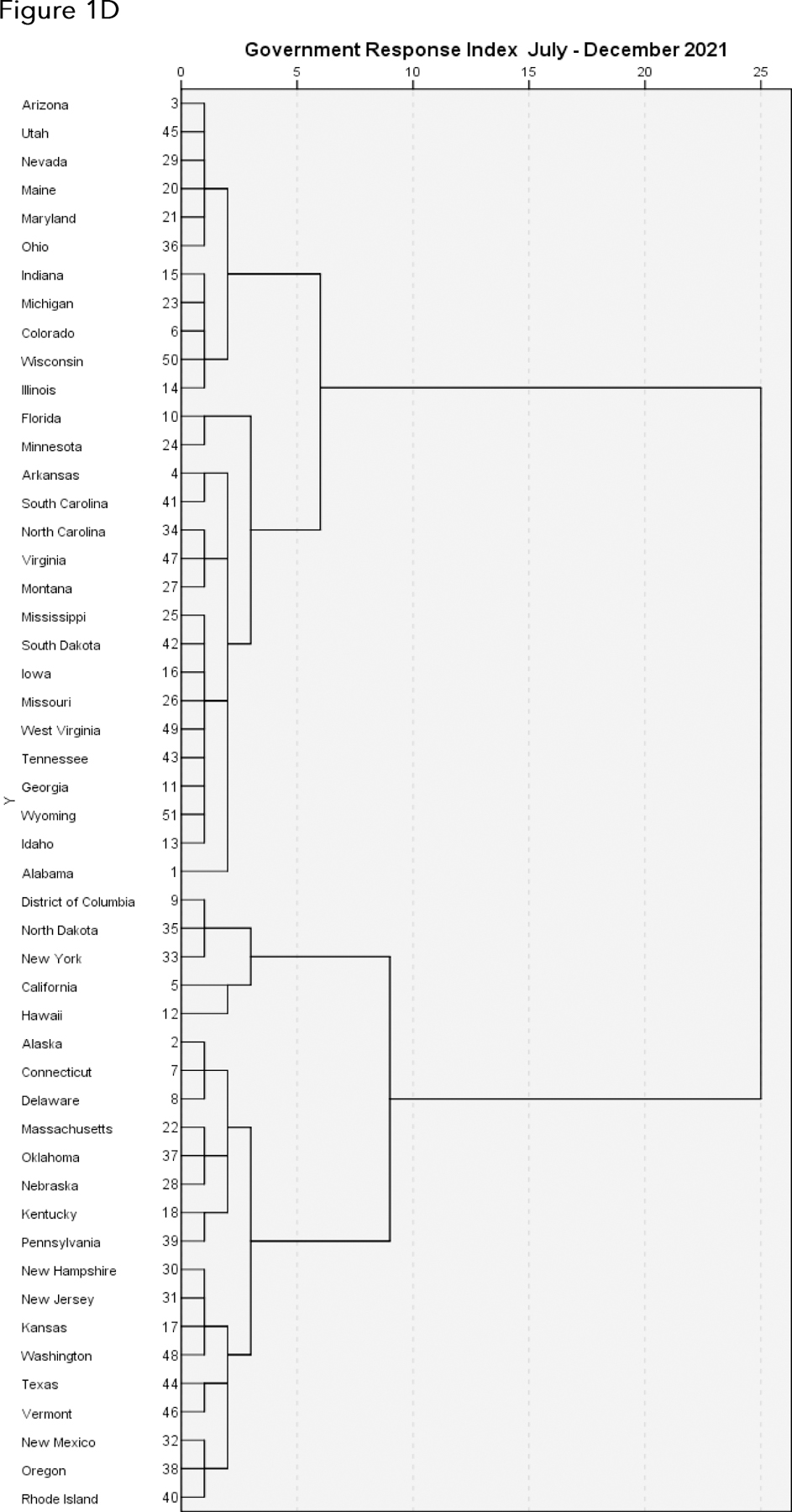

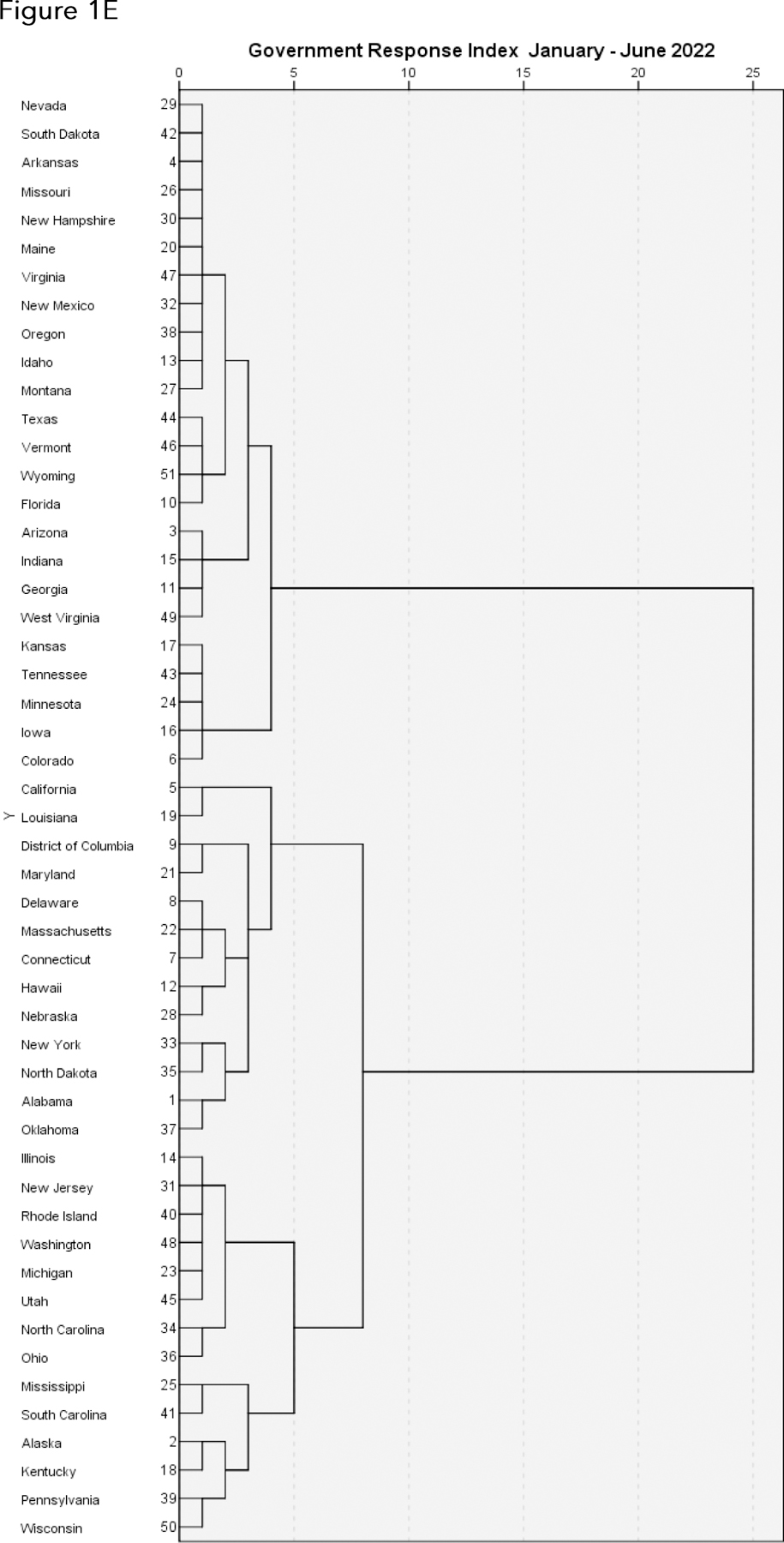
Dendrograms illustrating the arrangement of clusters of US states and the District of Columbia over consecutive 6 month periods from January 2020 through Jun 2022, based on their daily scores on the Oxford Government Response Index (GRI), a measure of their responses to the ongoing COVID-19 pandemic. The clusters are constructed from the Ward method of hierarchical cluster analysis, with a Minkowski metric for determining degrees of similarity between the states relative to the GRI. Viewers should focus on the horizontal distance at which any two states are joined together: the smaller the horizontal distance of the link that joins two states, the more similar the states are relative to their GRIs over that time frame. The numbers adjacent to the states’ names along the left margin are the states’ positions alphabetically from A to W. A. January through June 2020. B. July through December 2020. C. January through June 2021. D. July through December 2021. E. January through June 2022.

Dendrograms of the clusterings of states relative to the Oxford Government Response Index (GRI) are shown in Figure 1, separately for each 6 month period between January 2020 and June 2022. [As described in Methods, we utilized the Ward method of hierarchical cluster analysis, together with a Minkowski metric for numerical distances between the states relative to the GRI.]

**Figure 2.**
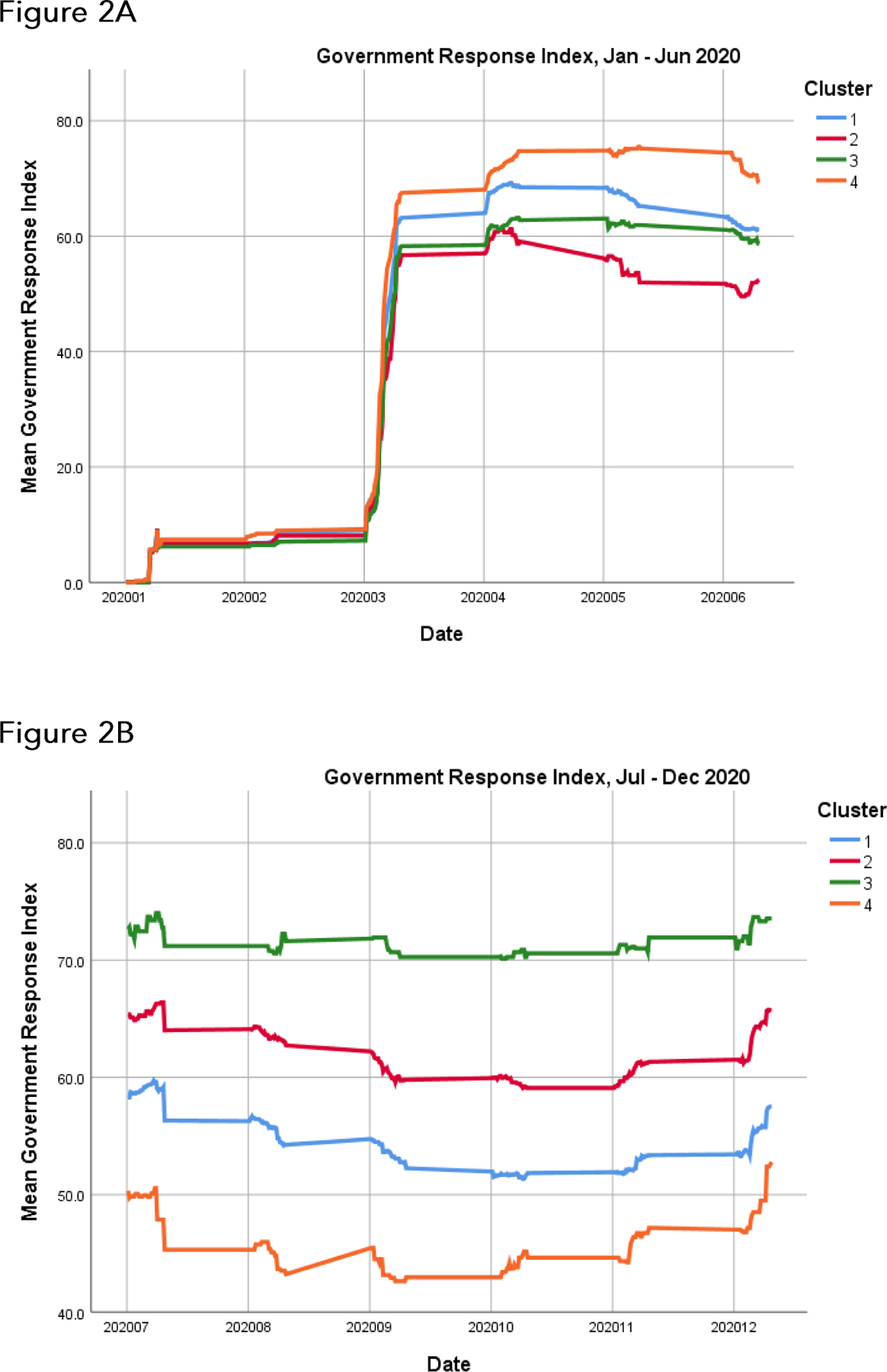

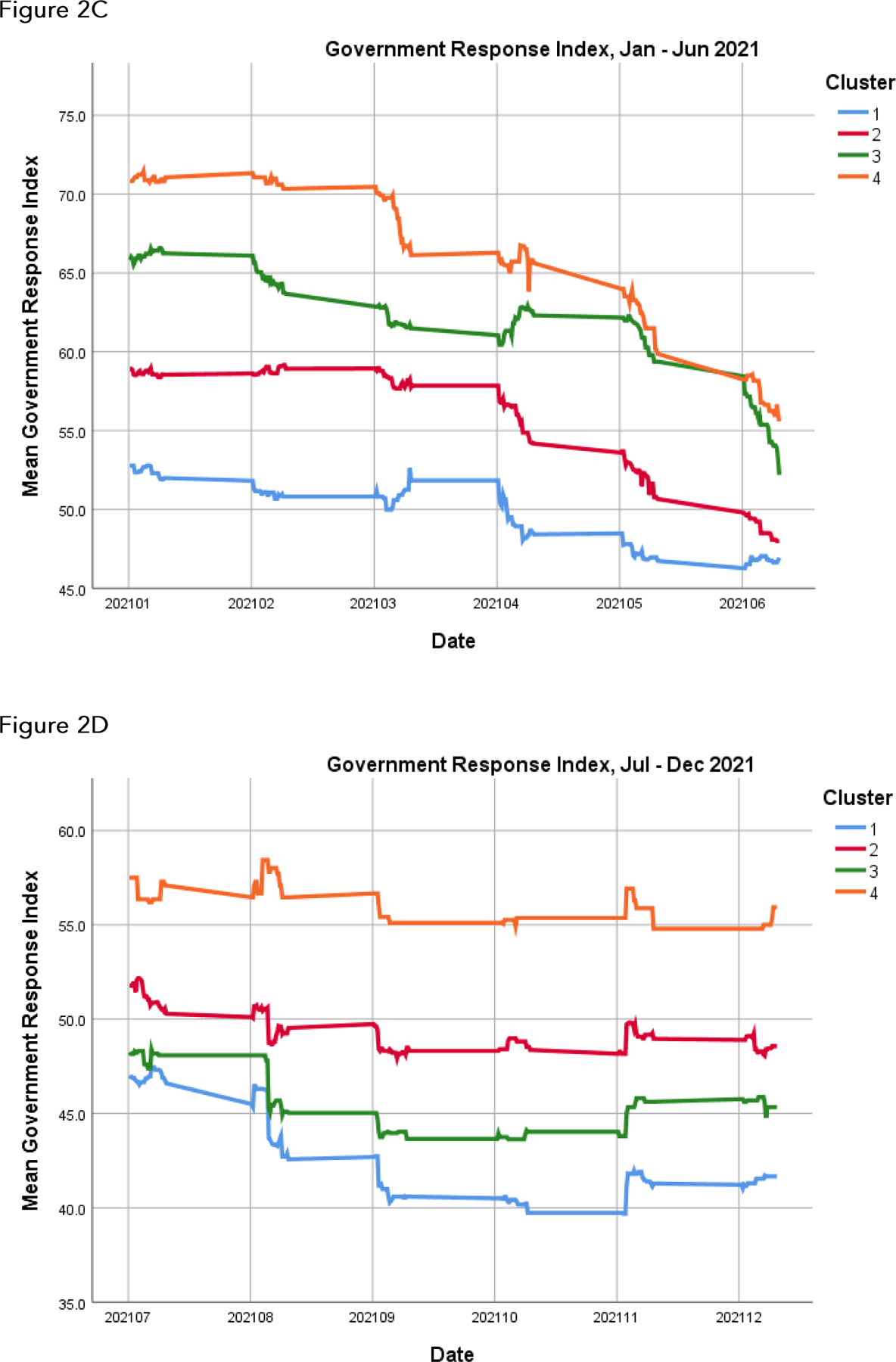

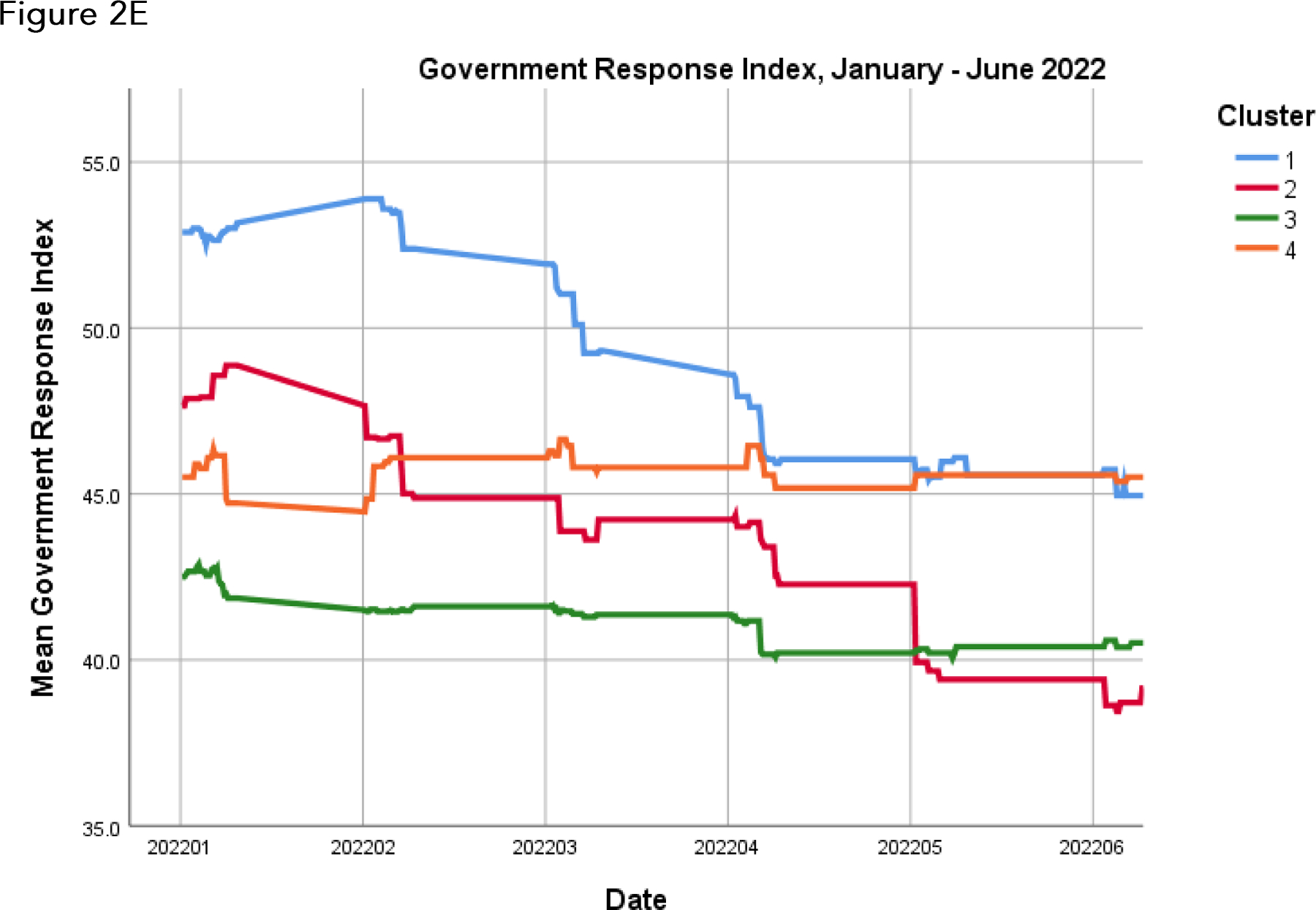
Average Government Response Indices in each of the four summary groups of states or clusters most similar to one another within each group, as determined from the dendrograms. The four clusters identified from the dendrograms represent mutually exclusive but relatively homogeneous groups of states, in decreasing order of similarity. A. January through June 2020. B. July through December 2020. C. January through June 2021. D. July through December 2021. E. January through June 2022. The x-axis labels denote year (2020, 2021, or 2022) followed by month (01 through 12).

**Figure 3.**
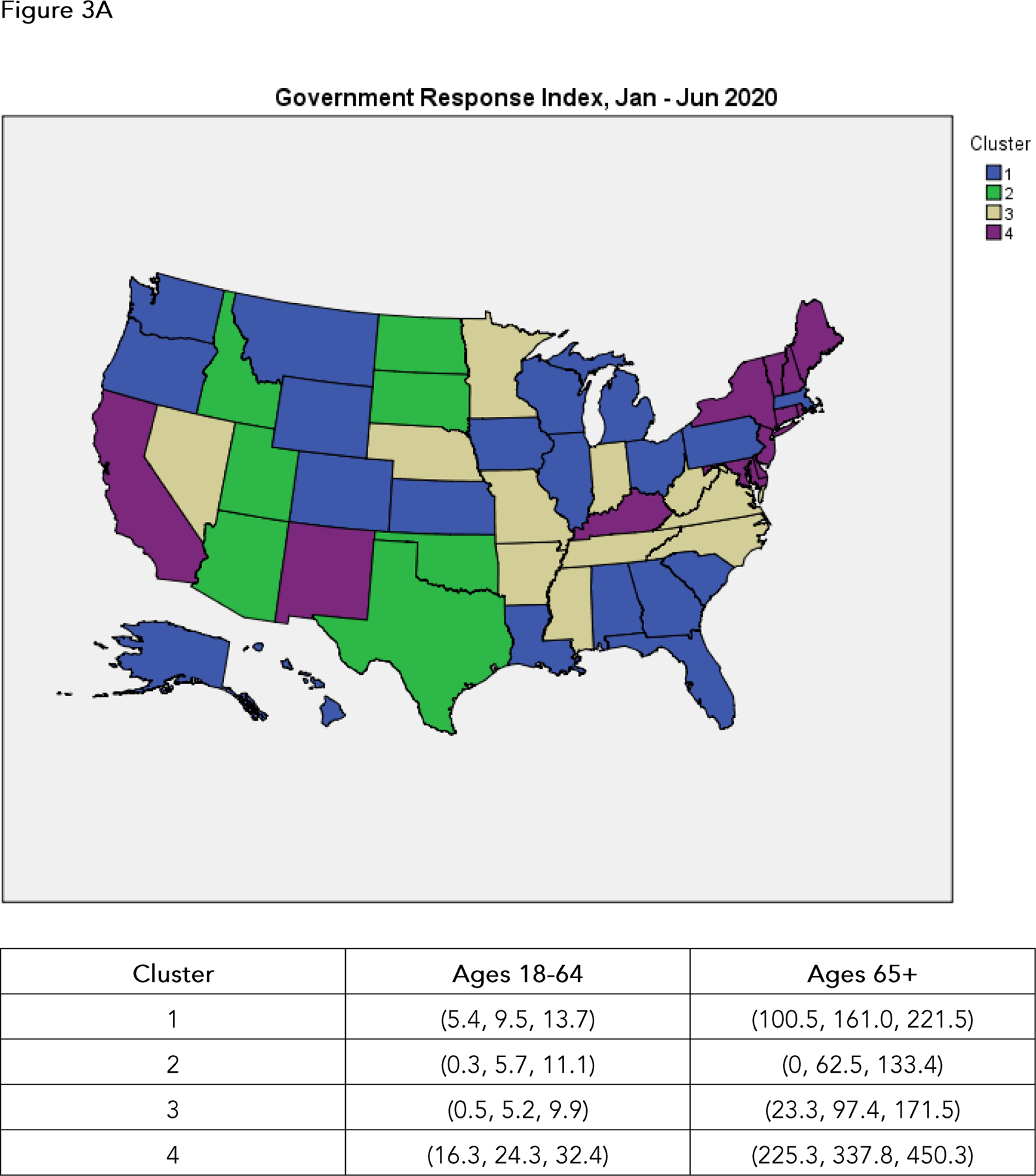

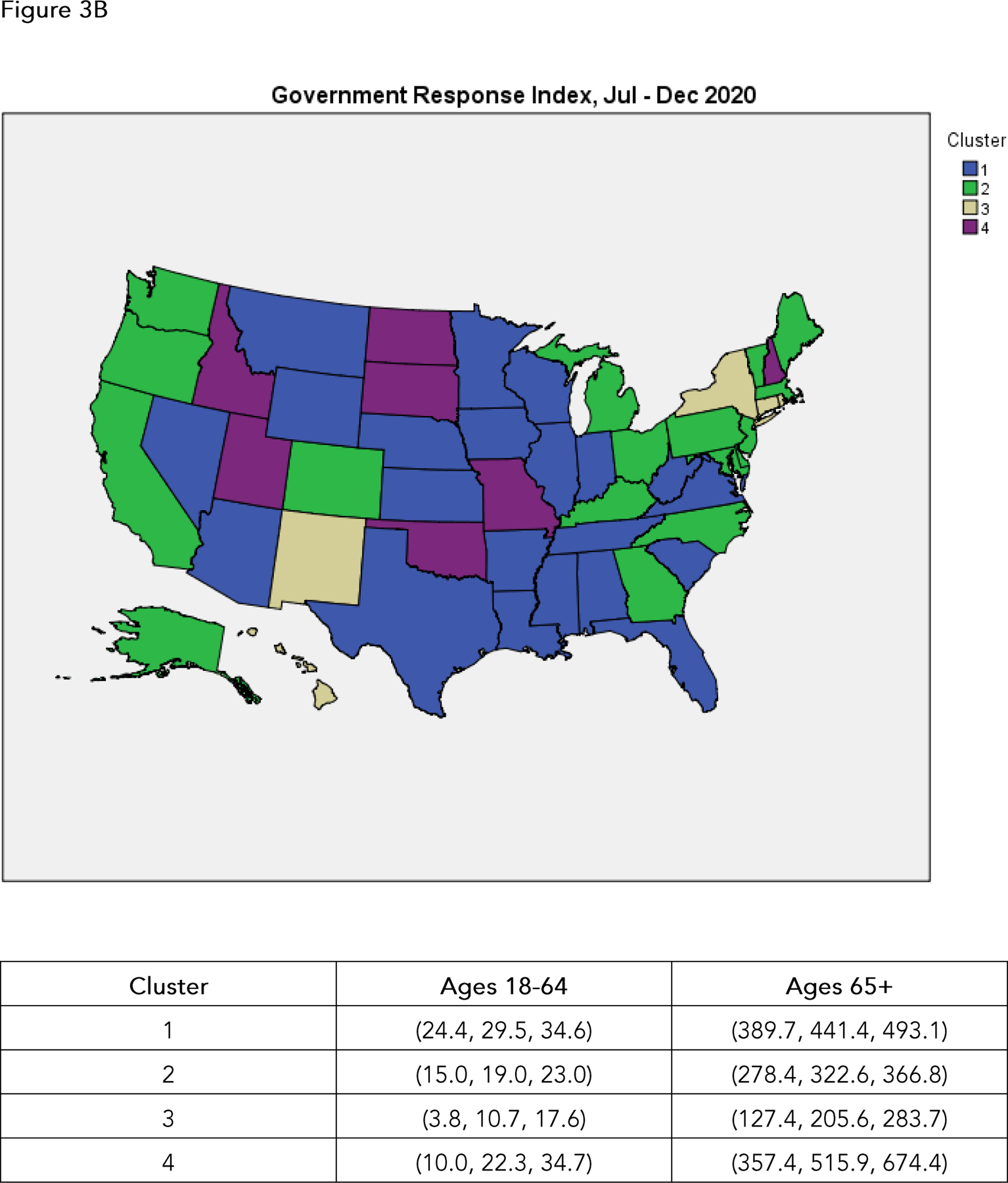

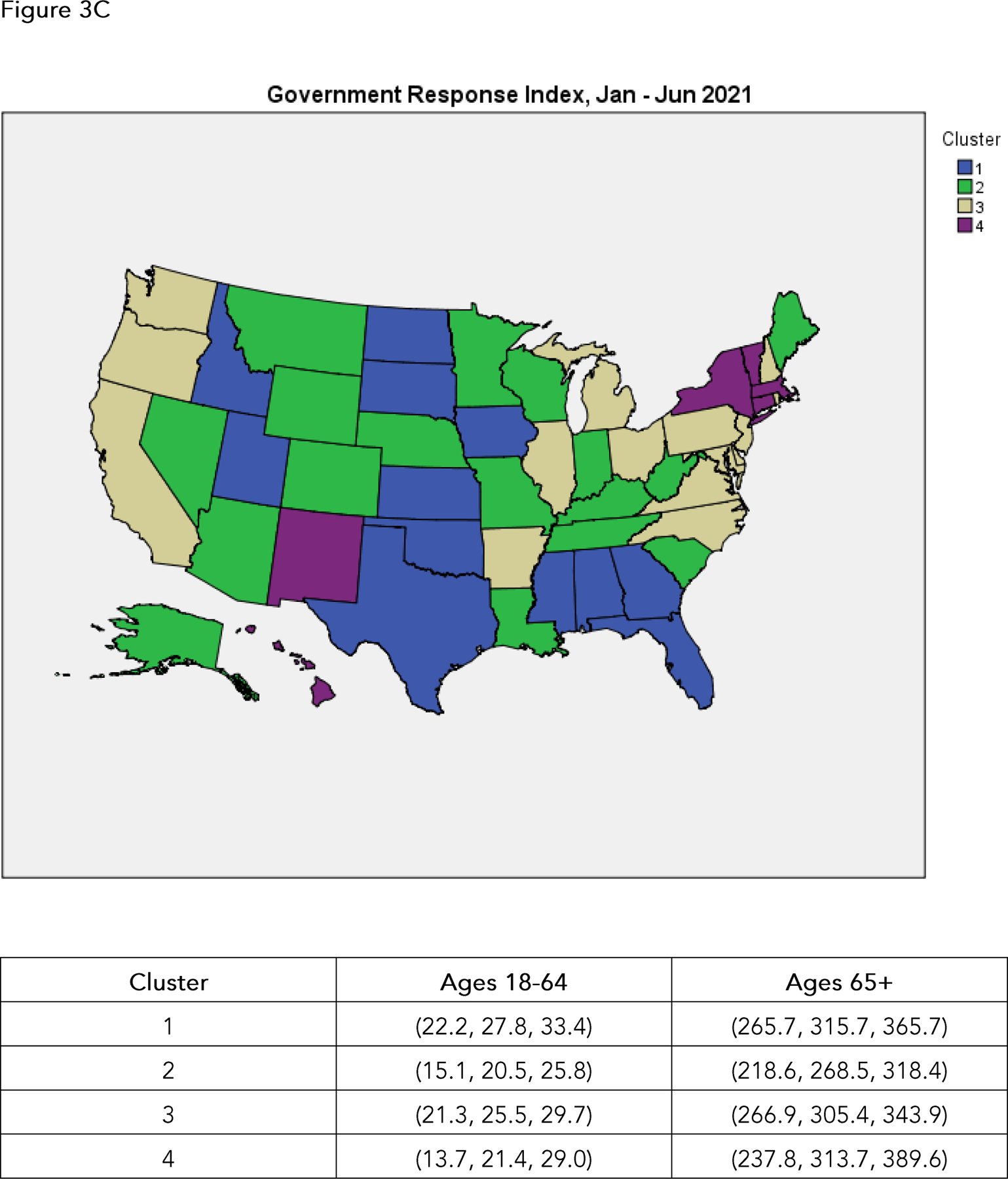

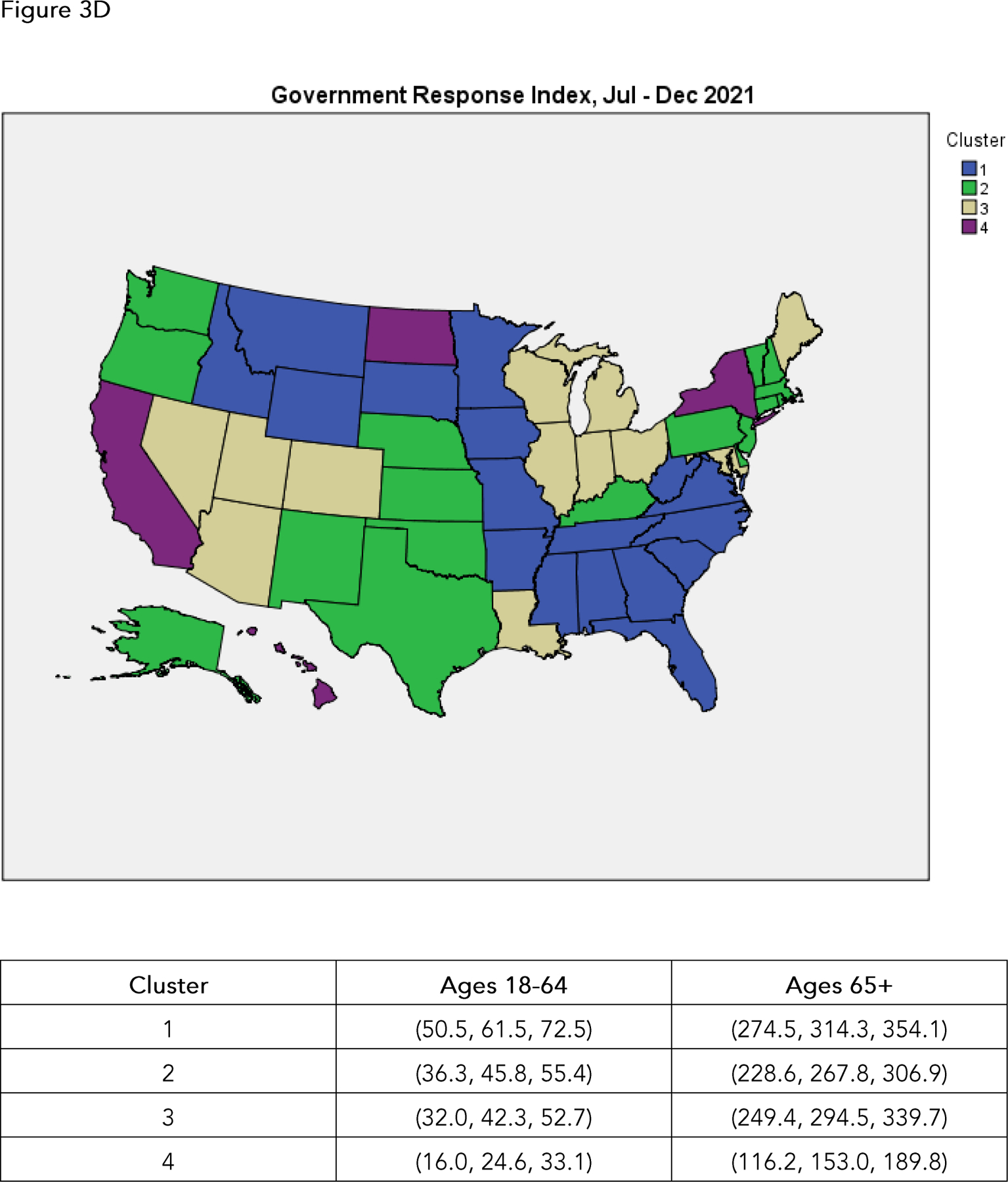

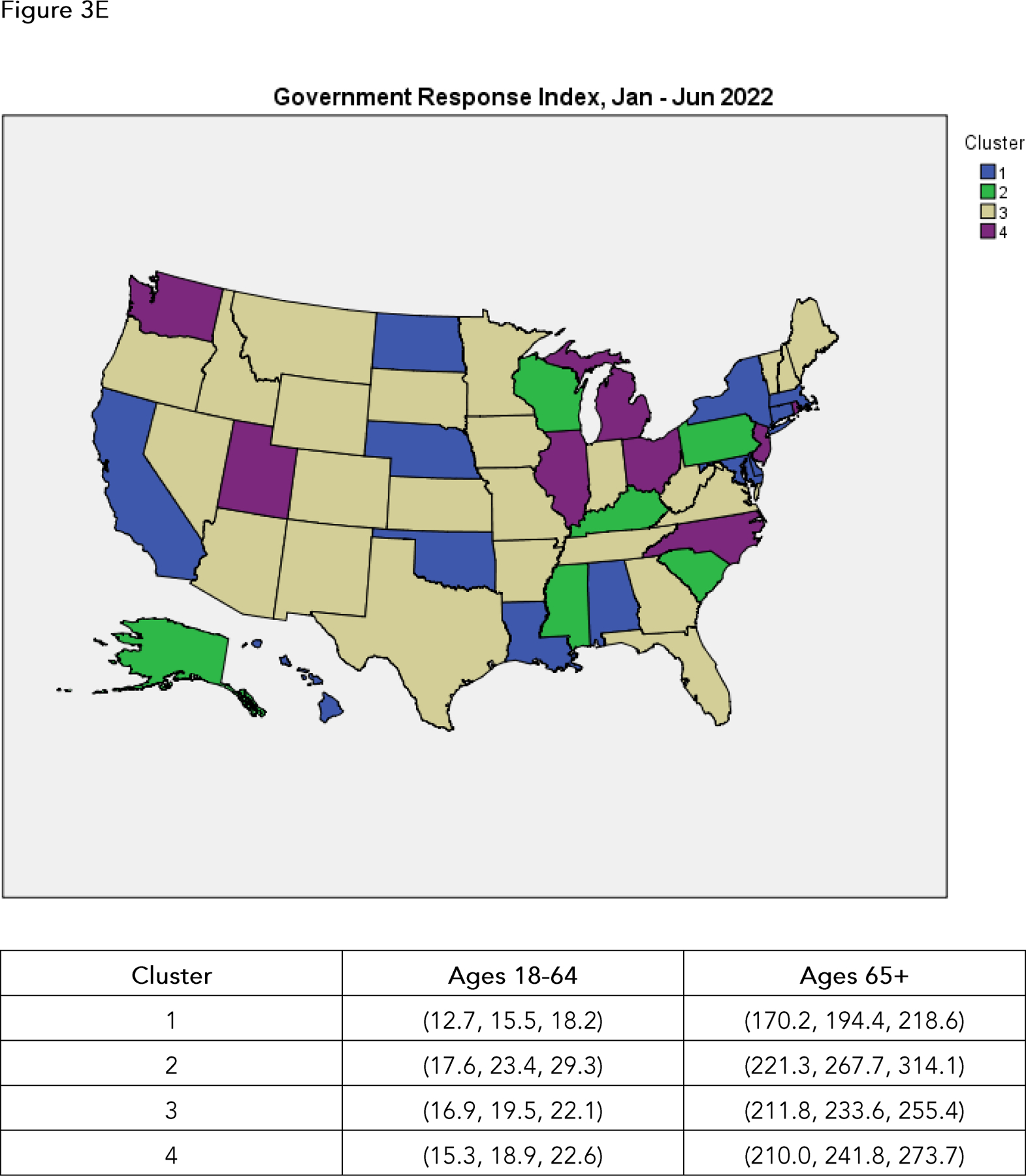
Choropleths illustrating membership in each of the four summary groups or clusters of states as described previously. The tables below the choropleths give estimated mortality rates and associated 95% confidence intervals (lower confidence limit, estimate, upper confidence limit) in each of the four clusters of states, separately for individuals ages 18 to 64 years old and individuals 65 years and older, as described in the Methods. A. January through June 2020. B. July through December 2020. C. January through June 2021. D. July through December 2021. E. January through June 2022.

From the hierarchical cluster analysis algorithm as depicted in each dendrogram, we combined clusters into four relatively homogeneous groups or clusters of states corresponding to their levels of agreement on the GRI over each 6 month period. For each of the five {year, period} pairings, we then plotted average GRI for each of the four subgroups of states (summary clusters), and also plotted choropleths to illustrate cluster membership (Figures 2, 3). We used Poisson regressions to estimate mortality rates and associated 95% confidence intervals for each of the four clusters of states within each {year, period} pairing, separately for individuals aged 18 to 64 and individuals 65 years and older. These estimates are given underneath the choropleths in Figure 3.

With regard to the plots of GRI over time (Figure 2), the index appears to be rather constant over each time frame in 2020 and 2021 across the four clusters of states, but with two notable transitions: the precipitous rise in GRI in March 2020, and the gradual decline in GRI during April through June of 2021. Following this gradual decline, the GRI was sustained at a much lower level over July through December 2021 compared to the previous year. The relative concordance across states relative to their GRIs in 2020 and 2021 was followed by discordance in early 2022 (Figure 2E): the 2022 experience does not mirror the previous two years.

Within each {year, period} pairing, mortality rates in 18 to 64 year olds across the four clusters of states generally tracks that of the elderly population (65 years +), though at a much lower level. Overall, there is little concordance between GRI levels and mortality rates. During the January - June 2020 period, the ordering of mortality rates in the four state clusters (lowest to highest) is the inverse of the ordering of the clusters in terms of levels of GRI (lowest to highest). During the July - December 2020 period, this pattern reverses: as state stringency increases as reflected in GRI, the mortality rates tend to decrease. No clear patterns of association between GRI scores and mortality rates are apparent in January - June 2021, but the July - December 2021 period is somewhat similar to July - December 2020, with more stringent GRI scores associated with lower mortality rates. In the January - June 2022 time frame, the states with the most stringent GRI scores experienced the lowest mortality rates in both the adult and elderly age groups; but the relative orderings of the remaining clusters of states in terms of both GRI scores and mortality rates is uncertain, and grouping the 2022 experience with that of the previous two years seems somewhat imprudent.

**Figure 4.**
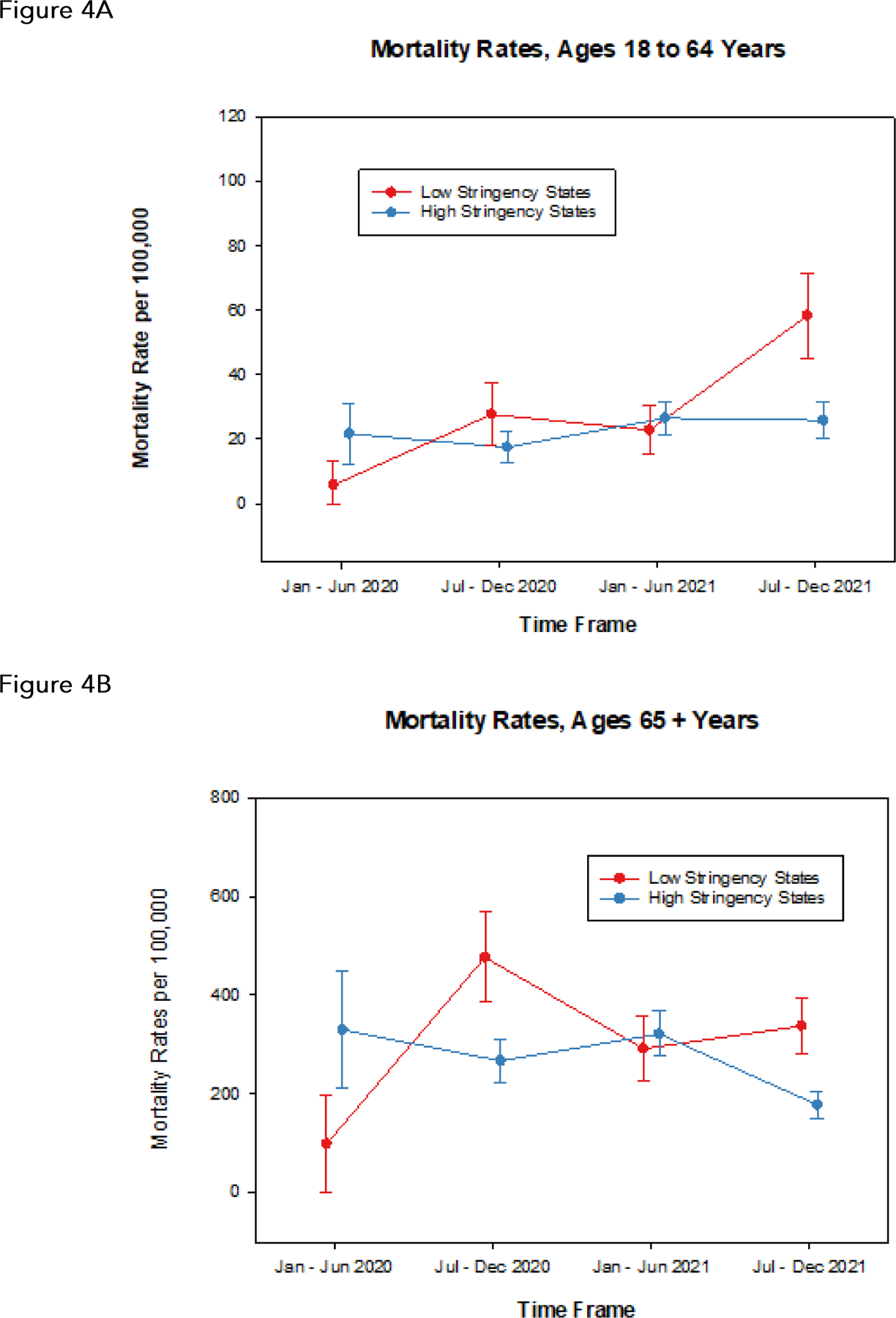
Estimated mortality rates and associated 95% confidence intervals over time for two subgroups of states: those that were consistently in the top two (most restrictive) tiers over 2020 and 2021 from Figure 2, and those that were consistently in the bottom two (least restrictive) tiers. The most restrictive states were: California, Connecticut, Delaware, District of Columbia (not a state), Hawaii, Massachusetts, New Jersey, New Mexico, New York, Oregon, Pennsylvania, Rhode Island, Vermont, Washington. The least restrictive states were: Arizona, Idaho, Indiana Minnesota, Mississippi, Missouri, Nevada, South Dakota, Tennessee, Utah, West Virginia. A Ages 18 to 64 years. B. Ages 65 years and older.

From the data underlying Figure 2, we found that 14 states were consistently in the top (most restrictive) two tiers of GRI across all four 6 month periods between 2020 and 2021: California, Connecticut, Delaware, District of Columbia (not a state), Hawaii, Massachusetts, New Jersey, New Mexico, New York, Oregon, Pennsylvania, Rhode Island, Vermont, Washington. We also examined whether states were consistently in the bottom (least restrictive) two tiers over the same time frame. These states were: Arizona, Idaho, Indiana, Minnesota, Mississippi, Missouri, Nevada, South Dakota, Tennessee, Utah, West Virginia, 11 in total. New York is an exemplar, always in the most restrictive cluster, as is South Dakota, always in the least restrictive cluster. We again used Poisson regressions to estimate mortality rates and associated 95% confidence intervals for these two subgroups of states, for each {year, period} pairing, and separately for the age groups 18 to 64 and 65 plus. These are displayed in Figure 4. Patterns are similar to our previous observations: during January through June of 2020, mortality rates are higher in the high stringency states compared to the low stringency states; this association is inverted during July through December of 2020 and again during July through December of 2021; patterns are similar in the two age cohorts 18 to 64 and 65 +, though with much lower mortality rates in the former age cohort.

**Figure 5.**
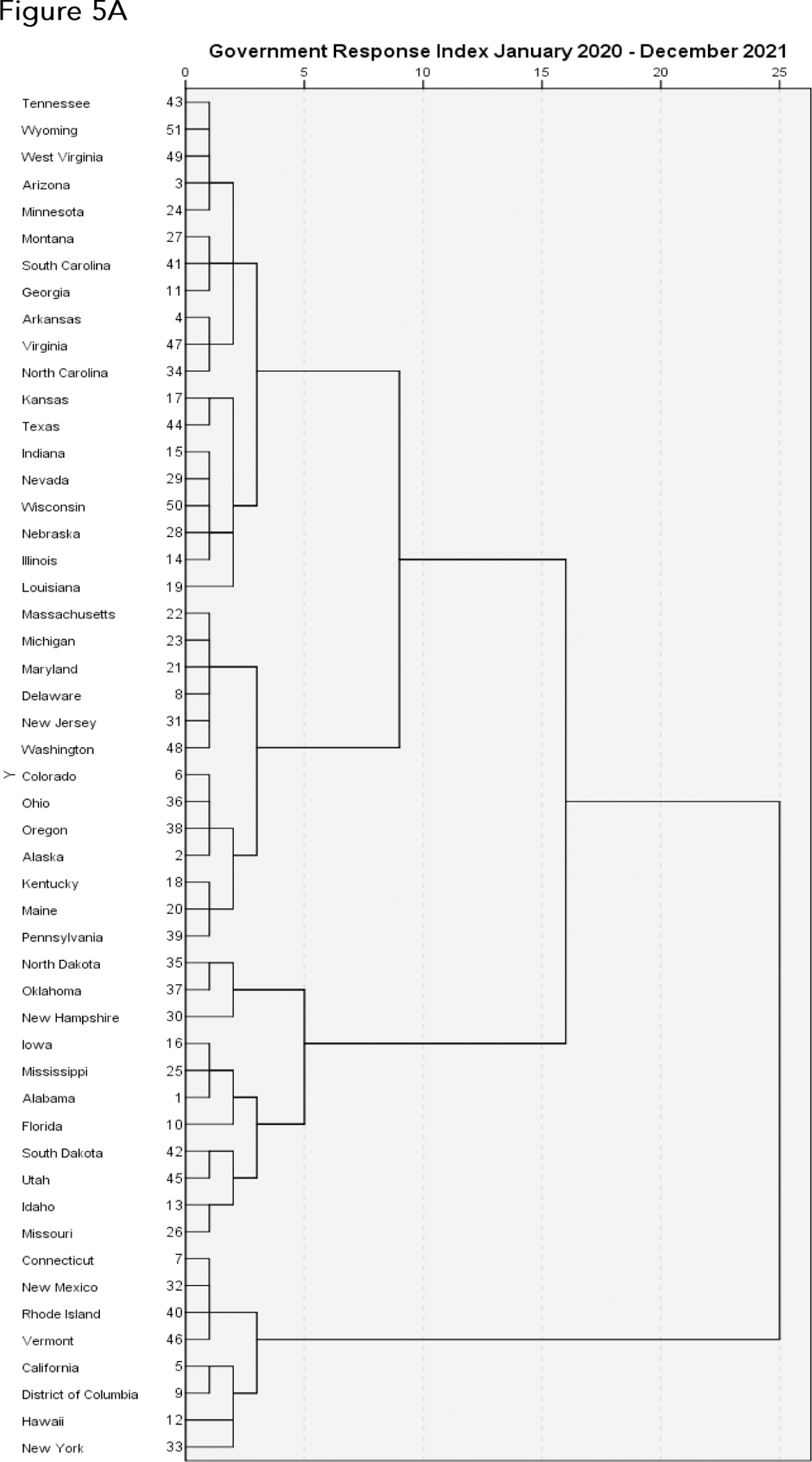

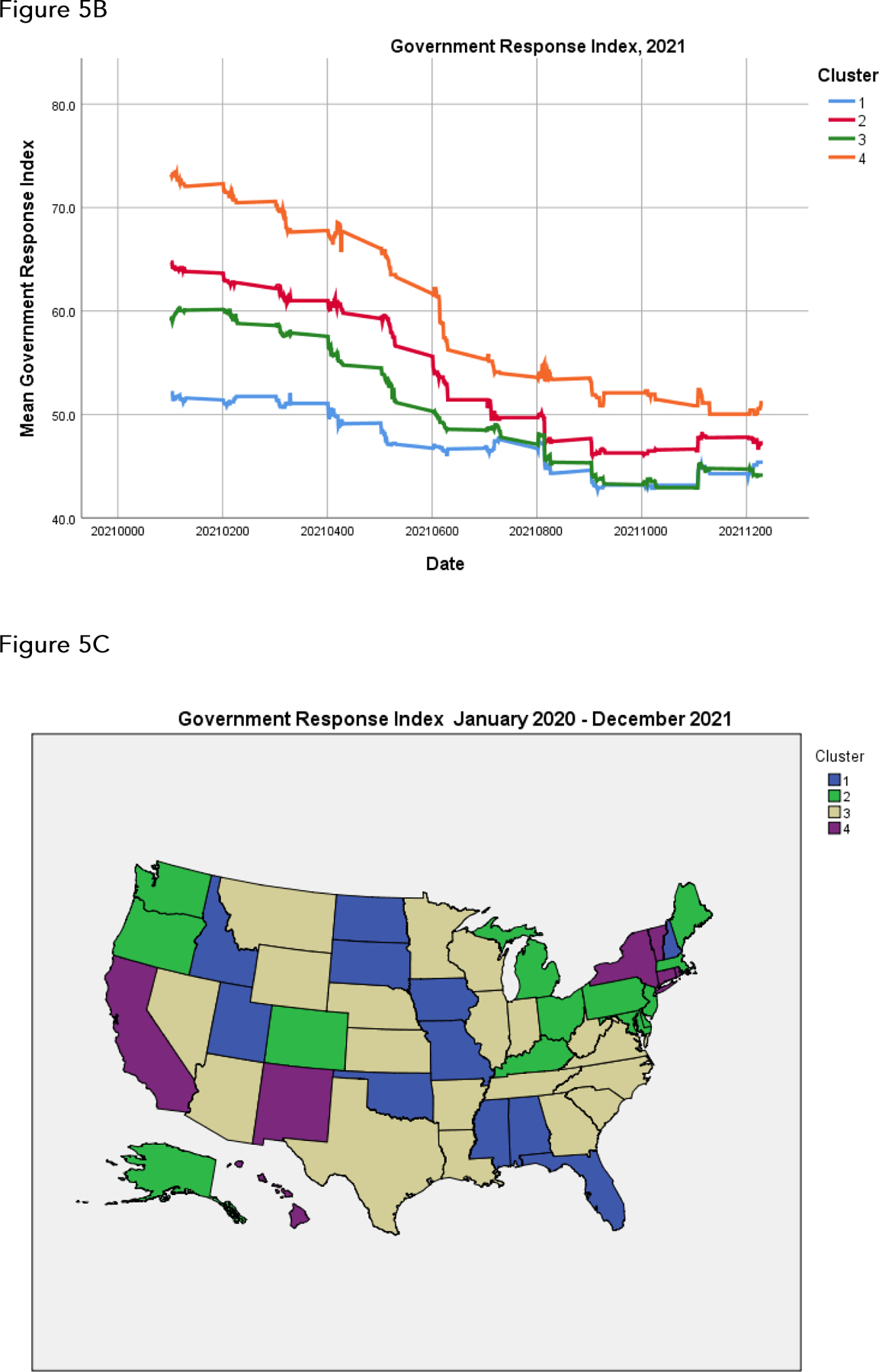

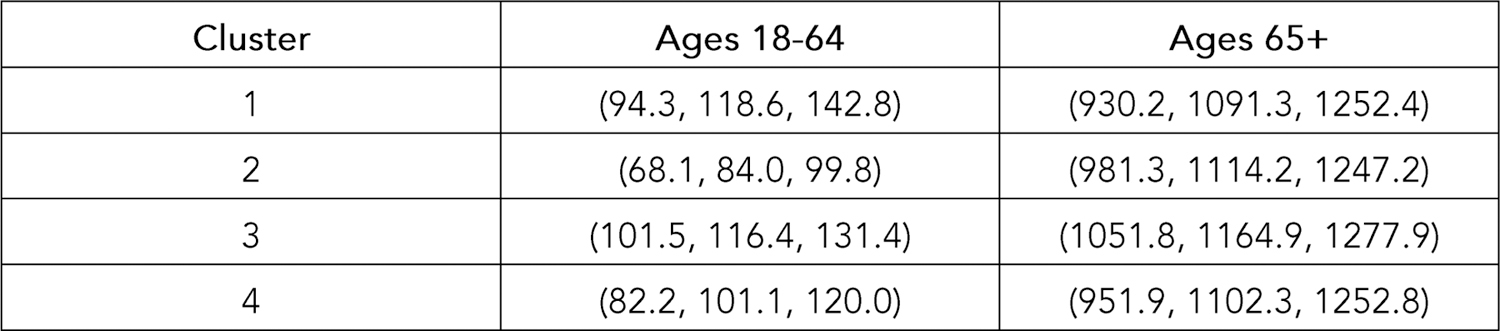
Dendrograms (A), Government Response Indices overtime (B), and chloropleths and mortality rates (C) for all states and the District of Columbia, over the two year time frame January 2020 - December 2021.

Lastly, we replicated the above analyses over the entire January 2020 - December 2021 time frame. Results are shown in Figure 5. The cluster representing the most restrictive tier of states, designated Cluster 4 in Figure 5, comprises California, Connecticut, District of Columbia, Hawaii, New Mexico, New York, Rhode Island, Vermont. The cluster representing the least restrictive tier of states, designated Cluster 1 in Figure 5, comprises Alabama, Florida, Idaho, Iowa, Mississippi, Missouri, New Hampshire, North Dakota, Oklahoma, South Dakota, Utah. In this overall representation, there are few substantial differences in mortality rates across the clusters, in either age cohort 18 to 64 or 65 +.

## DISCUSSION

Our study contributes to the expanding corpus of research dealing with the interplay between the public health impact of COVID-19 and government interventions, already summarized in (somewhat controversial) meta-analyses^7^. We focused on the Oxford Government Response Index, the most comprehensive policy index presented in the Oxford Government Response Tracker. As described in detail^2^, the GRI is comprised of 16 sub-indices: 8 indices for closures and containment, 2 for economic support, and 6 reflecting health measures. The Oxford Containment and Health Index and the Oxford Stringency Index are also in the Oxford database; both are subsets of the GRI, with slightly less comprehensive coverage at 14 and 9 sub-indices respectively. Our utilization of the Oxford database to probe the interplay between COVID-19 and government interventions is not unique^8,9,10^, and the influence of the Oxford database and similar endeavors is growing^11,12^.

Our investigation is closely related to a previous report^13^, also based on the OxCGRT data. That working paper encompassed a more limited time frame, the last months of 2020 through the first 100 days of the Biden administration in 2021, a period that included the nationwide dramatic decline in stringency criteria (Figure 2). The authors found substantial variation both within and between states relative to COVID-19 policy responses, which they documented by delving more intensively into individual components that comprise the Government Response Index (in particular, mask mandates, gatherings and events, back to work or school, vaccination rollout policies). We build on their findings by extending the time frame to encompass all of 2020 and 2021 experience, and by incorporating mortality data into state comparisons.

We found that there is not necessarily a strong association between stringency as reflected by the GRI and mortality attributable to COVID-19. One might expect that increased government stringency would be associated with lower COVID-19 mortality, but that notion is readily dispelled during the initial phase of the COVID-19 pandemic, January through June 2020. In isolation, this observation likely contributed to the rather tendentious conclusions of some investigators^7^. The initial COVID-19 wave during January through June of 2020 was largely absent from a broad swath of the United States, and the states initially hard-hit by the wave were the first to impose rigorous public health measures in reaction to the wave. The expectation that increased government stringency is associated with reduced COVID-19 mortality seems met only during the periods July - December 2020 and July - December 2021. We nevertheless emphasize that we are reporting associations, not causation, so that any evidence of government policy effects on COVID-19 mortality is at best suggestive, not conclusive.

The notable drops in stringency levels during May through June of 2021 coincides with the widespread rollout of vaccines, and were sustained at these lower levels during the subsequent 6 month period July - December 2021, compared to the 2020 experience. Unfortunately, there was no corresponding decrease in mortality during the July - December 2021 period, except, perhaps in the elderly (ages 65 and over), residing in the most stringent tier. One might argue that the May - June easing of restrictions throughout the country (e.g., easing of mask restrictions or social distancing; nearly complete abandonment of testing or case tracking) and the nationwide downplaying of the impact and severity of COVID-19 was perhaps premature, at least from a public health perspective. We also remark that there had been a substantial level of federal government support (e.g., extended unemployment benefits, small business loans or grants) were available in all states, so that the GRI had a raised floor until cessation of these programs (roughly September 2021 for extended unemployment benefits, December 2021 for small business loans). On the other hand, the majority of policy action occurred at the state or local level, with the federal role generally adopting an advisory role, limited to recommendations for states to adopt (or ignore).

The comparison of mortality rates in high stringency states to low stringency states (Figure 4) points to a national divide on a number of levels: obviously, political, red states (Republican leaning) vs. blue states (Democratic leaning)^14, 15^; but also geographical, the Northeast and West coasts vs. the interior (Midwest, South, far West)^16^; or, overall population densities in predominantly rural vs. predominantly urban states^17^. A virtually identical pattern was reported in the Oxford study^13^, over the first 100 days of the Biden administration; it is noteworthy that this demarcation has persisted over two years, not merely 100 days. Nevertheless, close scrutiny of our choropleths reveals that the state clusters are not identifiable solely on the basis of political leanings, geographical location, or population density: similarities or dissimilarities across states cross these boundaries.

We remark that, dependent on the time frame used, one might easily conclude there is no evidence of any effects of government policy on COVID-19 mortality: for example, the January - June 2020 experience, viewed in isolation, depicts increasing stringency associated with increasing mortality rates. Our examination of the January 2020 - December 2021 time frame en masse (Figure 5) found few substantive differences in COVID-19 mortality rates across the different stringency tiers of states, in either age cohort. This two year time frame should subsume geographical waves of infection, and the emergence and evolution of more lethal variants of SARS-CoV-2. [Notably, mortality rates have gradually diminished with the appearance of Omicron and subsequent variants in late 2021.] One might well seize upon this finding as evidence writ large that stringency measures were of little practical benefit. We remark that, in this overall two-year comparison, Vermont, which maintained higher stringency over time, evinced the lowest observed mortality rates among all states both for the elderly (65 and older: about 193 per 100,000 population) and the middle-aged (aged 18 through 64: about 5 per 100,000 population). In stark contrast, the highest observed mortality rates were experienced by Mississippi: about 182 per 100,000 among the middle-aged, and about 1530 per 100,000 among the elderly. The two states have quite different stringency profiles, but there are other factors at play: the population of Vermont is predominantly white, and the state enjoys favorable geographical characteristics for weathering pandemics; Mississippi, in contrast, has a large minority population, and there are disconcertingly large racial and ethnic disparities in COVID-19 mortality (among other salient factors) in the United States^18^. The disproportionate impact of COVID-19 on communities of color and low-income individuals reflects stark inequities in the US public health system, already apparent over a century ago in the 1918 flu epidemic^18^. Federal investment in and the coordination of the United States public health system has been lacking^19^; and, state and local public health programs have been chronically underfunded^20^. Political trends and motivations are also at play, of course^21,22^, as personal responsibility cannot always overcome inadequate government response. In this regard, the discordant 2022 findings might be attributed to a number of factors, such as increased vaccination rates in certain states, an easing of government strictures, a general weariness with governmental accounts of the ongoing severity of the pandemic, and the intangible factor of individual compliance with public health measures and mandates.

We categorize our analyses as exploratory, and acknowledge a number of limitations. The Oxford Government Response Index tracks mandated behavioral changes (e.g., lockdowns, school closures), but not voluntary behavioral changes. Enforcement and compliance are not tracked; and it is apparent that two years into the pandemic, social exhaustion with restrictions and a greater acceptance of risk are widespread throughout the country. Furthermore, vaccination status and immunity rendered from previous infections will affect mortality data, though perhaps not incidence. As noted previously, timing is a fundamental issue: causality follows from temporality, which is difficult to discern in this observational study: indeed, at the state or local level, stringency measures are often introduced in response to spikes in incidence. Importantly, the reported mortality numbers and rates are unadjusted for key risk factors, such as obesity, hypertension, and diabetes. The underlying hierarchical cluster analyses (Figure 1) are intrinsically dependent on the choice of methodology (here, Ward clustering) and distance metric (here, Minkowski) for determining clusters, based on the states’ daily scores on the Government Response Index. Identical results to those presented here if based on different cluster algorithms or distance metrics should not necessarily be expected. Also, our amalgamation of states into four groups or clusters over each {year, period} pairing (Figures 2, 3) generally appears to be a straightforward consequence of the observed separation of clusters in Figure 1, but again might not be reproducible with other cluster algorithms or distance metrics. In short, given the exploratory nature of cluster analysis, we hesitate to impute any statistical significance to this methodology. On the other hand, we believe our analyses are largely in accord with previous findings (13); they remain informative and of practical significance, as they provide germane insights into states’ variability in their responses to the COVID-19 pandemic in 2020, 2021, and 2022, and concomitantly in their COVID-19 mortality rates.

### Conclusions

State governments in the United States have manifested considerable heterogeneity relative to stringency requirements related to public health, imposed in response to the COVID-19 pandemic. Nevertheless, evidence that increased governmental stringency at the state level is associated with lower COVID-19 mortality is equivocal. Political trends and motivations, together with a shifting social climate, appear to have an outsized influence on governmental responses to the COVID-19 public health crisis.
